# Structural morphing in a symmetry-mismatched viral vertex

**DOI:** 10.1038/s41467-020-15575-4

**Published:** 2020-04-06

**Authors:** Qianglin Fang, Wei-Chun Tang, Pan Tao, Marthandan Mahalingam, Andrei Fokine, Michael G. Rossmann, Venigalla B. Rao

**Affiliations:** 1 Department of Biological Sciences, Purdue University, West Lafayette, IN 47907 USA; 2 Department of Biology, The Catholic University of America, Washington, DC 20064 USA

**Keywords:** Phage biology, Virus structures, Cryoelectron microscopy

## Abstract

Large biological structures are assembled from smaller, often symmetric, sub-structures. However, asymmetry among sub-structures is fundamentally important for biological function. An extreme form of asymmetry, a 12-fold-symmetric dodecameric portal complex inserted into a 5-fold-symmetric capsid vertex, is found in numerous icosahedral viruses, including tailed bacteriophages, herpesviruses, and archaeal viruses. This vertex is critical for driving capsid assembly, DNA packaging, tail attachment, and genome ejection. Here, we report the near-atomic in situ structure of the symmetry-mismatched portal vertex from bacteriophage T4. Remarkably, the local structure of portal morphs to compensate for symmetry-mismatch, forming similar interactions in different capsid environments while maintaining strict symmetry in the rest of the structure. This creates a unique and unusually dynamic symmetry-mismatched vertex that is central to building an infectious virion.

## Abstract (web summary)

In icosahedral viruses, a symmetry-mismatched portal vertex is assembled by inserting a 12-fold-symmetric portal complex into a 5-fold-symmetric capsid environment. Here, the authors report a near-atomic-resolution in situ cryo-electron microscopy structure of this symmetrically mismatched viral vertex from bacteriophage T4.

## Introduction

Symmetry mismatches in macromolecular assemblies are ubiquitous in biological systems^1^. It introduces flexibility and dynamism that are fundamentally important for executing biological functions. Examples include viruses and numerous cellular complexes including, cytoskeletons, secretion systems, and so on. Of these, the viral portal vertex represents an extreme form, and probably the most widely distributed symmetry-mismatched structures in the biosphere^2,3^. It is universally found in tailed bacteriophages (phages), herpesviruses, and archaeal viruses, and is structurally well-conserved^4–6^. Phylogenetic analyses indicate that it is one of the most ancient structures, dating back to an early time in evolution when self-replicating entities appeared on Earth^7^.

The viral portal vertex consists of a 12-fold-symmetric dodecameric portal complex inserted into a 5-fold-symmetric capsid vertex, making it the unique vertex of an icosahedral viral capsid^2,8^. It is an extreme form of symmetry mismatch where the sub-assemblies, the portal and the capsid, have completely different structures, symmetries, and functions. It is also the first-assembled virus structural unit, which nucleates capsid (head) assembly^9^. Next, by interacting with a motor protein, it assembles a powerful packaging machine that translocates viral genome through its ~35 Å central channel^4,6^. Sensing the fullness of genome-packaged head, the portal vertex then expels the packaging motor and binds to head-tail connector proteins that seal off the head and attach an infection machine such as the tail. When infection occurs, the portal vertex acts again, releasing the viral genome into a new host cell through the same channel that is now aligned with a tail tube^10,11^. The symmetry-mismatched viral vertex, therefore, is central to driving key assembly steps that build a virus particle of precise dimensions, as well as its infectivity. However, despite more than five decades of intensive research and debate since discovery in 1965^12–14^, the structure of the symmetry-mismatched viral portal vertex, the unique interactions at the interface of capsid and portal, and how these interactions drive virus morphogenesis remained mysterious. Even the most recent high resolution cryo-electron microscopy (cryo-EM) structures of phages or herpesviruses could not resolve the symmetry-mismatched interface^15–17^.

The tailed bacteriophage T4 which infects *Escherichia coli* bacterium is one of the most well-characterized viruses, an exceptional model system in molecular biology, and a powerful platform for gene, vaccine, and therapeutic molecule delivery^18–20^. It consists of a large, prolate, icosahedral capsid (head), 1200-Å-long and 860-Å-wide that encapsidates 172-kbp double-stranded DNA genome. A 1400-Å-long contractile tail is attached to the unique portal vertex of the capsid. The capsid shell is made of the major capsid protein, gp23*, which is organized into a hexagonal lattice characterized by triangulation numbers T_mid_ = 20 for the elongated midsection and T_cap_ = 13 laevo for the two icosahedral caps^21^. The outer surface of the shell is decorated by two nonessential proteins, Hoc and Soc^22,23^. Eleven vertices of the capsid are occupied by pentamers of gp24*^24^, whereas the unique twelfth vertex, the portal vertex to which the packaging motor or tail are attached, is occupied by the dodecameric portal protein, gp20^7,25^.

The very first step in phage T4 morphogenesis is the assembly of gp20 portal protein into a dodecamer on the cytoplasmic surface of the inner *E. coli* membrane^9,26^. Early genetic studies showed that the portal dodecamer nucleates head assembly by interacting with the major capsid protein gp23 and the major scaffolding core protein gp22^27,28^. This nucleating nexus then goes on to build the capsid shell around the scaffolding core which also incorporates several other core proteins including the capsid maturation protease gp21^29^. Capsid maturation ensues, with the gp21 protease cleaving the N-terminal 65-residues and 10-residues of gp23 and gp24 to generate gp23* and gp24*, respectively, and degrading the scaffolding core to small peptides, which leave the capsid and create room for genome packaging^9,30,31^. The “empty” capsid is released from the cell’s membrane into the cytosol for DNA packaging. A pentameric packaging motor then attaches to the portal and fills the capsid with the phage genome^32^. Capsid expansion accompanies DNA packaging leading to large conformational changes in the major capsid protein and an increase in capsid volume by ~50%. The packaging motor dissociates from the portal when the head is full while the gp13-gp14 neck complex associates, sealing off the packaged head^31^. The tail then attaches to the neck complex to complete virus assembly. The initial interactions between the dodecameric portal and the gp23 capsid protein are, thus, central to the creation of the symmetry-mismatched vertex, which then sets the stage for the entire virus assembly.

Here, we report a high-resolution in situ structure of a symmetry-mismatched viral portal vertex from bacteriophage T4, which illuminates the key interactions at the interface of the 5-fold capsid and 12-fold portal. Further, the in situ structure uncovers an extraordinary viral adaptation—structural morphing—where the local structure of the portal subunits morphs, reaching out to nearby capsid subunits and forming similar interactions in different capsid environments. Unlike the rigid-body motions commonly associated with conformational transitions, these structural morphing events engage only the local regions of the portal’s wing domain that faces the capsid while the rest of the portal structure remains in strict 12-fold symmetry. Our genetic analysis shows that a key morphing region, the N-terminal whisker, anchors the portal to the capsid and is essential for capsid assembly and phage viability. Besides compensating for structural and symmetry conflicts, structural morphing creates an unusually dynamic vertex that drives key, distinct, and competing, viral assembly processes. This might be a general and fundamental phenomenon in viruses and many other biological systems.

## Results

### In situ structure of the phage T4 portal vertex

To determine the in situ structure of phage T4 portal vertex, we produced mature empty capsids and collected large cryo-EM datasets using a Titan Krios microscope equipped with a K2 Summit direct electron detector (Supplementary Fig. 1a). Three cryo-EM reconstructions of the T4 portal region were calculated using different symmetries: a 12-fold-averaged reconstruction of the portal protein; a 5-fold-averaged reconstruction of the gp23* capsomers surrounding the portal; and an asymmetric reconstruction of the portal and surrounding capsomers, showing the portal-gp23* interface. The resolutions of these reconstructions were 3.8, 3.4, and 4.5 Å, respectively, determined by the gold-standard FSC cutoff of 0.143^33^ (Supplementary Fig. 1b–f). These reconstructions were used to build the atomic structure of the portal protein and five surrounding gp23* capsomers (Supplementary Table 1, Supplementary Fig. 1c–e).

**Fig. 1:**
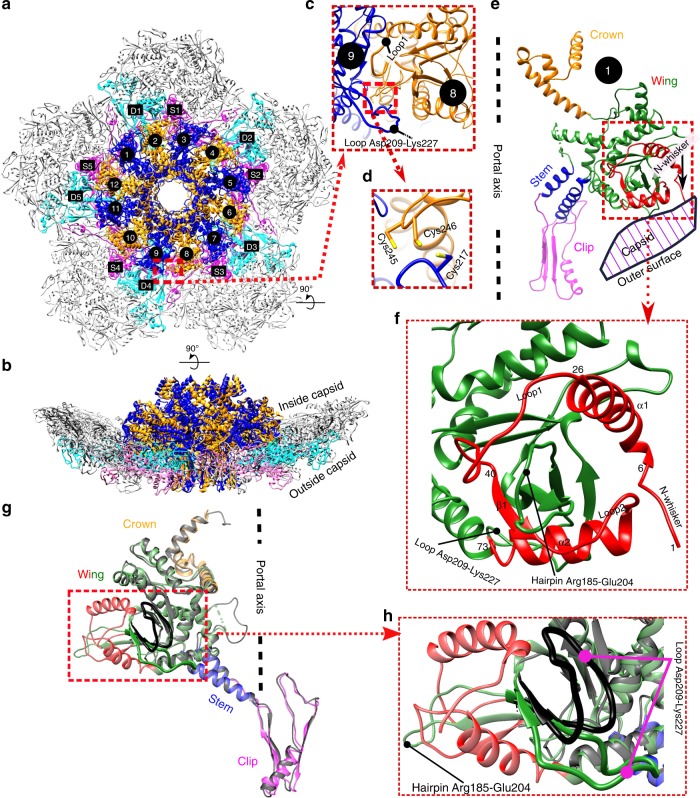
In situ structure of the portal assembly and the surrounding capsomers. **a**, **b** Overall structure, top and side views. The gp20 portal subunits are colored blue and orange and numbered 1 to 12. The S subunits (numbered S1 to S5) and D subunits (numbered D1 to D5) of the major capsid protein gp23* are colored magenta and cyan, respectively. **c**, **d** Inter-subunit interactions of the portal assembly. The enlarged inset shows the possible site for inter-subunit disulfide bonding. **e**, **f** Structure of gp20 subunit 1. The crown, stem, and clip domains are colored orange, blue, and magenta, respectively. The N-terminal 73 residues and other regions of the wing domain are colored red and green, respectively. The N-whisker region in **e** has been indicated by a black arrow. **g**, **h** Comparison of the in situ gp20 structure with the previously reported recombinantly expressed N-terminal deletion gp20 structure (PDB: 3JA7). The in situ gp20 structure (subunit 1) colored as in **e** is superimposed on the recombinant gp20 structure colored gray. The bent loop of the recombinant portal structure is shown in black.

The in situ portal structure has the shape of a flying saucer with a central channel for DNA entry and exit (Fig. 1a, b and Supplementary Movie 1). Each gp20 subunit consists of a crown domain, a wing domain, a stem domain, and a clip domain (Fig. 1e). Although the overall in situ portal structure is similar to our previously reported structure of the recombinant N-terminal deletion gp20 mutant^7^ (with an RMSD of 1.6 Å between 4740 corresponding Cα atoms, or 80% of the in situ portal), the two structures show major differences, which also define the structure and function of this symmetry-mismatched viral vertex (Fig. 1c–h). First, the 73-residue-long N-terminal portal region, which was missing in the recombinant portal structure but now fully resolved in the asymmetric in situ portal vertex reconstruction, is critical for attachment of the portal to the capsid (Fig. 1f, h; red). Second, two loop regions of the portal wing domain, hairpin Arg185-Glu204 and loop Asp209-Lys227, which make critical contacts with the capsid, display different conformations in the in situ and recombinant structures (Fig. 1h). Importantly, the above regions exhibit “morphing” in the in situ structure to accommodate the symmetry mismatch (see below).

**Fig. 2:**
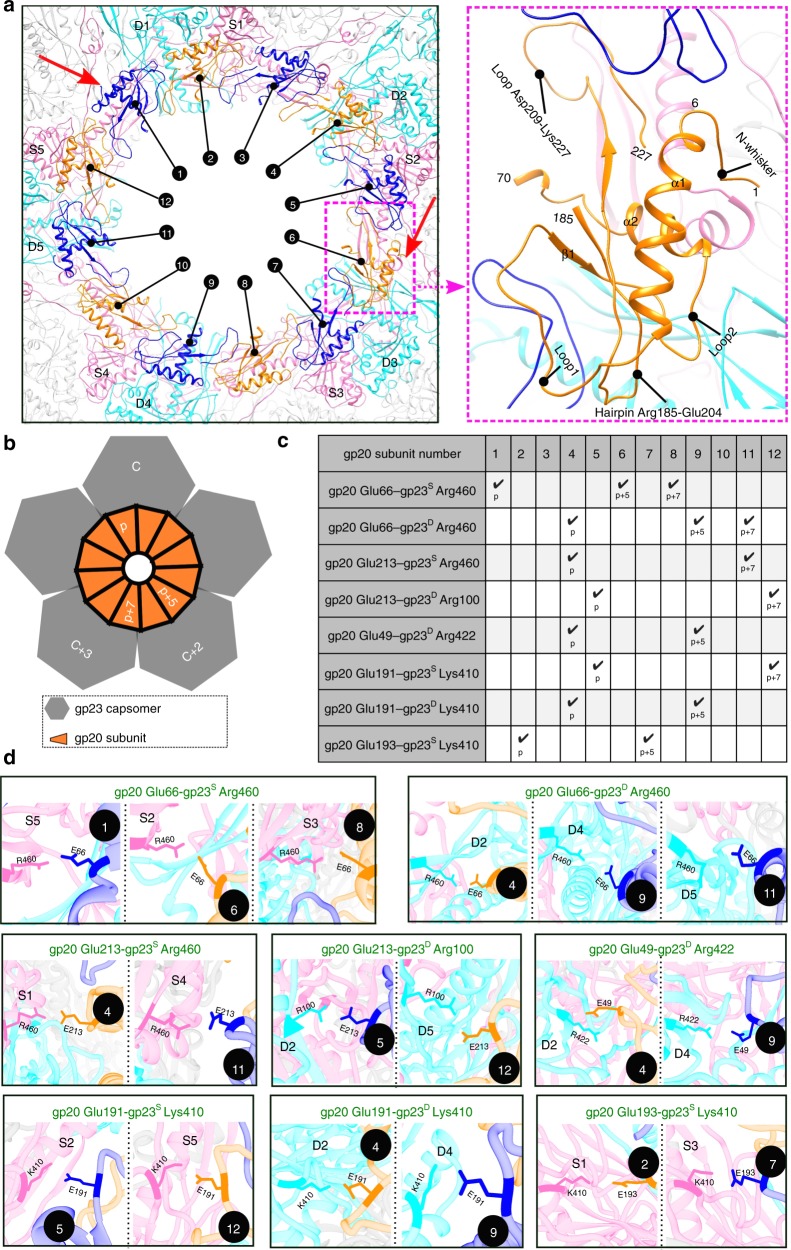
Portal-capsid interactions in the symmetry-mismatched vertex. **a** Arrangement of the portal and capsid subunits in the in situ structure. The gp20 subunits and gp23* subunits are colored and labeled as in Fig. 1a (S and D gp23* subunits in magenta and cyan, respectively, and alternative gp20 subunits in blue and orange). For clarity, only the N-terminal 70 residues, loop Asp209-Lys227, and hairpin Arg185-Glu204 of each gp20 subunit that make contacts with the capsid are shown and one of the subunits is enlarged. The two potential methionine-metal clusters are indicated by red arrows. **b** Schematic diagram showing the symmetry mismatch between the portal assembly and the capsid. **c**, **d** Charge interactions between the portal and its surrounding gp23* capsomers showing a morphing relationship between p, p + 5, and p + 7 portal subunits to compensate for the symmetry mismatch.

The newly-resolved N-terminal region is located inside the mature capsid and enlarges the wing domain of the portal (Fig. 1e, f). It starts with a flexible 6-amino acid N-whisker (residues 1–6), which is followed by α-helix1 (Fig. 1f). This helix is connected to a short β-strand1 by a 15-residue-long loop1, which is involved in extensive interactions with a neighboring gp20 subunit (Figs. 1c, 2a). The β-strand1 is followed by a flexible loop2 and α-helix2. These β-strand, loops, and helices interact with and stabilize the 19-residue-long hairpin Arg185-Glu204 (Fig. 1f), which is disordered in the recombinant portal structure but well-resolved in the in situ structure. This hairpin together with the N-terminal region interacts with the gp23* capsomers in situ (Fig. 2a). In addition, the flexible gp20 loop Asp209-Lys227 that follows the hairpin adopts a bent conformation in the N-terminal deletion mutant (Fig. 1h; black), whereas in situ, it swings downwards with its Cys217 residue at the crown of the loop placed in close proximity to Cys245 and Cys246 residues of the adjacent gp20 subunit on its “right” (Fig. 1d, h). This could possibly allow the formation of inter-subunit disulfide bonds between Cys217 of one subunit and Cys245 or Cys246 of the adjacent subunit. The above networks of inter-subunit interactions greatly strengthen the in situ structure of the portal dodecamer.

### Structural morphing of portal subunits

**Fig. 3:**
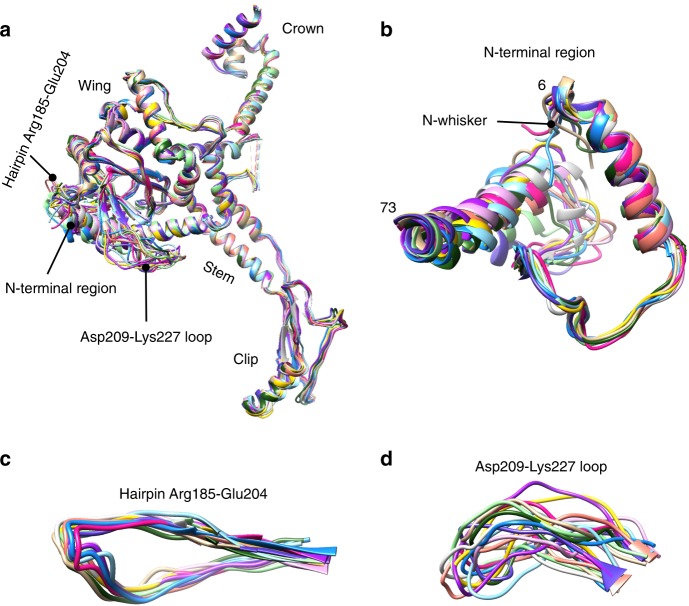
Superimposition of all the twelve gp20 subunits within the portal assembly. **a** The gp20 subunits. **b** N-terminal region of gp20 subunits. **c** Hairpin Arg185-Glu204 of gp20 subunits. **d** Asp209-Lys227 loop of gp20 subunits.

Although most of the in situ portal dodecamer structure obeys 12-fold symmetry, its periphery does not, as it morphs at the points of contact with the capsid subunits. In total, the gp20 dodecamer interacts with ten subunits of gp23*, two from each of the five gp23* capsomers that encircle the portal (Figs. 1a, 2a; Supplementary Movie 1). These ten subunits can be divided into two types: five surrounding, or “S” gp23* subunits that form a continuous ring beneath the portal’s wing domain (Fig. 1a; magenta), and five distal, or “D” gp23* subunits that are more distant to portal (Fig. 1a; cyan). Strikingly, the overall structures of the gp23* subunits and five hexameric capsomers surrounding the dodecameric portal are similar to gp23* subunits surrounding the pentameric gp24* vertices of our previously reported icosahedrally-averaged capsid structure^34^. The gp23* subunits strictly obey the 5-fold symmetry and show no significant conformational differences except at the N-fist regions (residues 66–91) which are disordered in the five D subunits (Supplementary Fig. 2) because these N-fist regions now face the portal rather than a capsomer as in the rest of the capsid where they are stabilized by inter-capsomer interactions. The angles between the capsomer planes are also similar near the portal vertex and the gp24* vertices, indicating that the presence of the portal protein instead of gp24* does not induce significant changes in the capsid shell. In contrast, individual portal subunits undergo structural morphing and show large conformational differences in their N-terminal regions, Arg185-Glu204 hairpins, and Asp209-Lys227 loops (Fig. 3). For example, when the portal subunits 1 and 6 are superposed, the distance between the C-alpha atoms of residues Lys2, belonging to N-whiskers, is 6.5 Å (Supplementary Fig. 3). Another example, when the portal subunits 3 and 7 are superposed, the distance between the C-alpha atoms of residues Ala224, located in loop Asp209-Lys227, is 12 Å (Supplementary Fig. 3).

Because the capsomers surrounding the portal do not exhibit significant conformational differences, each subunit of portal dodecamer in the symmetry-mismatched vertex finds itself in a different capsid environment (Fig. 2a). However, subsets of portal subunits encounter somewhat similar capsid environments because of the rotational symmetries of the portal and the capsid shell (Fig. 2b). Indeed, the portal structure repeats at 30° intervals, α_1_ = p*30°, while the capsid structure repeats at 72° intervals, α_2_ = c*72°, where p and c are integer numbers of portal and capsid subunits, and α_1_ and α_2_ are rotational angles of the portal and capsid around their symmetry axes, respectively. Consequently, for p + 5 and c + 2 subunits, the difference between α_1_ and α_2_ is only +6°, and for p + 7 and c + 3 subunits, the difference is only −6°. Therefore, if the portal subunit p faces a certain capsid environment, the subunit p + 5 will face the same structure, rotated by +6°, and the subunit p + 7 will also face the same capsid environment but rotated by −6° (Fig. 2b). Thus, subsets of portal subunits, which have the p, p + 5, p + 7 relationships, have similar environments. Our asymmetric reconstruction shows that the portal subunits form a series of salt bridges with the gp23* molecules (Fig. 2c, d). Remarkably, however, the portal morphs to form similar salt bridges with the capsid subunits, by adjusting the positions of the loop regions of p + 5 and p + 7 portal subunits relative to subunit p, and compensating for the +/−6° differences at these positions of contact. For example, the Glu66 residues of the portal subunits 1 and 6 are involved in similar salt bridges with the Arg460 residues of gp23*. For this to occur, the portal structure morphs locally (Supplementary Movie 2) such that Glu66 in these two portal subunits attains similar capsid environment. Such morphing occurs at each set of portal subunits related by p, p + 5, and/or p + 7 positions (Fig. 2c, d), even though the actual salt-bridge forming residues vary, thus depicting an extraordinary adaptation of portal’s face while maintaining symmetry in the rest of the structure. Analysis of other portal structures suggest that the N-terminal region and loops located in the wing domain periphery are also flexible, implicating that similar symmetry-mismatch compensation mechanisms are likely employed by other viruses^15–17,35,36^.

**Fig. 4:**
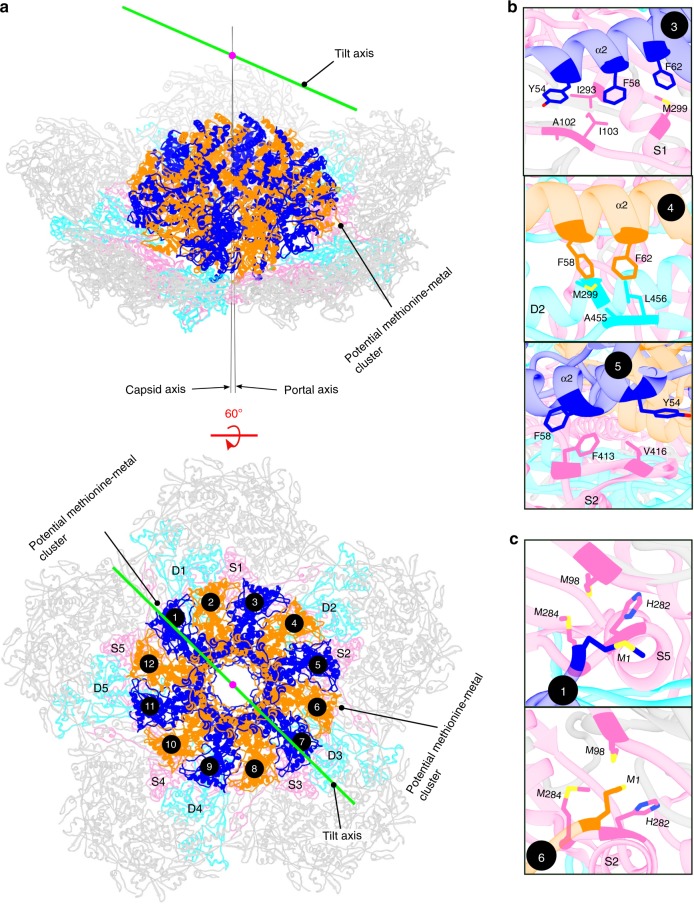
Tilt between the portal and the capsid and potential metal-binding clusters. The portal assembly and the surrounding gp23* capsomers are colored and labeled as in Fig. 1a. **a** Tilt between the portal and the capsid. The tilt axis, portal axis, and capsid axis are shown. **b** Hydrophobic interaction between gp20 α2 and gp23* capsomers. **c** The potential methionine-metal clusters formed between the N-whisker methionine (M1) of subunits 1 and 6 of gp20 and subunits S5 and S2 of gp23*. Residues involved in forming the metal binding sites are labeled.

In addition, the portal is anchored to the capsid at two points by morphing of two of the N-terminal whiskers (Supplementary Movie 3). The N-terminal methionines of the whiskers belonging to portal subunits 1 and 6, which also have the p, p + 5 relationship, form putative metal-binding clusters with the capsid subunits (Fig. 4a, c). Each cluster consists of Met1 of gp20, and Met98, His282 and Met284 from a gp23* S subunit. Met98 is located in the N-arm of gp23*, whereas His282 and Met284 belong to the gp23* P-domain^34^ (Supplementary Fig. 2). A similar cluster is also used by the CopC protein from *Pseudomonas syringae* to bind Cu^+^ ion with a K_D_ of 10^−13^ M^37^. Therefore, it is likely that the two clusters at the portal-gp23* interface coordinate with Cu^+^ ions and greatly reinforce the portal-capsid binding by anchoring the portal to the capsid.

### Portal is tilted relative to capsid

The in situ structure shows that the portal and capsid axes of the symmetry-mismatched vertex are misaligned, resulting in an unexpected ~0.9° tilt of the portal with respect to the capsid (Fig. 4a). The center of the portal rotation is located at approximately 130 Å above the portal’s center of mass (Fig. 4a). This portal tilt results in favorable hydrophobic interactions of α-helices2 of three portal subunits located on one side of the portal with the neighboring gp23* molecules (Fig. 4b). Notably, the tilt axis is roughly parallel to the line connecting the two anchor points implicating that the methionine-metal clusters might be involved in regulating portal-capsid interactions during assembly and genome packaging.

### The portal N-whisker is essential for capsid assembly

**Fig. 5:**
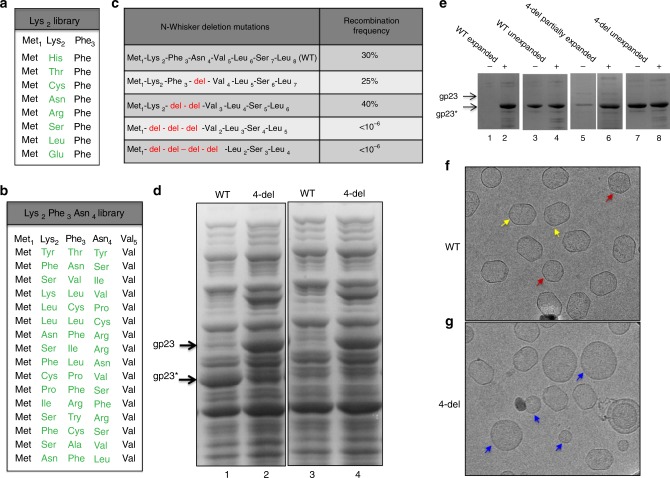
The N-whisker is essential for capsid assembly. **a**, **b** Functional single and three amino acid substitution mutants in N-whisker from CRISPR combinatorial libraries. **c** Frequencies of recombination that resulted in infectious phage using various deletion mutants in N-whisker. **d**, **e** SDS-polyacrylamide gel electrophoresis of lysates of infected *E. coli* cells (panel **d**) and purified proheads (panel **e**). Most of the major capsid protein gp23 was uncleaved in the 4-del portal mutant (lane 2) and remained (unassembled) in the supernatant upon high speed centrifugation (lane 4) whereas it was cleaved to gp23* which occurs only following capsid assembly (lane 1) and very little of it remained in the supernatant (lane 3). **e** Expanded, partially expanded, and unexpanded proheads purified by CsCl gradient centrifugation and DEAE ion-exchange chromatography. The samples were concentrated and approximately 2 × 10^10^ prohead particles were loaded in each lane. “+” and “−” represent with and without boiling, respectively, of the purified prohead samples isolated from the ion-exchange column. The expanded heads are stable and remain intact in SDS without boiling (lane 1; no gp23* enters the gel), whereas the unexpanded capsids dissociate (lane 3). The 4-del mutant showed a partially expanded peak (lanes 5–6) but no fully expanded prohead peak as in the WT (lanes 1–2) (Supplementary Fig. 5). Note the presence of small amounts of uncleaved gp23 present in the 4-del mutant proheads. **f**, **g** Cryo-EM images of the expanded heads purified from WT and 4-del mutant infections as shown in panel e. Yellow and red arrows identify prolate, icosahedral capsids in side and top views, respectively. Blue arrows identify round capsids of different sizes. See Methods for more details.

Extensive mutagenesis of N-whisker by CRISPR genome editing showed that numerous substitutions are tolerated at the amino acids following the N-terminal methionine (Fig. 5a, b), however the length of the 6-amino acid whisker is critical for function. Shortening the whisker by deleting one or two amino acids did not affect phage viability whereas three or four amino acid deletions resulted in lethality (Fig. 5c). In recombinational rescue experiments, infectious phages were recovered at a frequency of 25–40% in the case of one or two amino acid deletions, similar to that of the wild-type (WT) sequence, whereas no progeny phages were recovered for three or four amino acid deletions even when ~10^6^ phage were used for infection (recombination frequency, <10^−6^). The lethal phenotype of three or four amino acid deletion mutants was further verified by an independent genetic approach where a double amber mutation in N-whisker was rescued instead of the CRISPER-directed cleavage of N-whisker (Supplementary Fig. 5) (see Methods for details). This means that the length of the whisker but not its amino acid composition, is the key to anchor the portal to the capsid through structural morphing. Morphing can tolerate trimming of the whisker, up to ~7 Å, but not beyond. Biochemical analyses showed that the 4-amino acid deletion (4-del) mutant is defective in capsid maturation cleavages and assembled ~3–10 times fewer capsids. Much of gp23 in the 4-del mutant remained uncleaved and in soluble form (Fig. 5d and Supplementary Fig. 6). Even in the assembled 4-del capsids, expansion was affected (Fig. 5e, Supplementary Fig. 4 and Supplementary Fig. 6), and there are many round particles of different sizes (Fig. 5f), unlike the uniform size prolate icosahedral particles produced by the wild-type portal protein (Fig. 5g).

## Discussion

The above studies uncovered the structural morphing phenomenon, as the underlying mechanism in the creation of the symmetry-mismatched portal vertex in phage T4. It should be noted that the environments of portal subunits in the capsid-portal interface are not quasi-equivalent. In case of quasi-equivalence^38^, all protein subunits obey quasi-symmetry and are located in similar environments. In case of the symmetry-mismatched portal vertex, all portal subunits cannot have similar environments. Only subsets of portal subunits, which have the p, p + 5, p + 7 relationships, face similar capsid environments, rotated by ±6°. For example, such a subset is formed by the portal subunits 1, 6, and 8. These subunits adopt different conformations to compensate for the 6-degree differences. Another subset of subunits, formed by subunits 2, 7, and 9, also face similar environments, rotated by only ±6°. However, the environments of subunits from the two different subsets (1, 6, 8 and 2, 7, 9) are completely different. Indeed, subunits 1 and 2 face different environments, rotated by 360/12 = 30°. Therefore, the adaptive conformational differences between subunits 2, 7, and 9 vary from those observed between subunits 1, 6, and 8. Hence, the symmetry mismatch compensation requires larger and differential conformational variability under different structural environments, which we define as structural morphing.

Early genetic studies have established that the unique portal vertex is essential to nucleate capsid assembly^27,28^. The same vertex, later, is also essential for other key viral assembly processes including capsid maturation, genome packaging, and neck/tail attachment^39^. Capsid assembly is possibly initiated when one of the N-whiskers captures a gp23 molecule and forms the methionine-metal cluster. The wing domain morphs and forms salt bridges with the captured molecule, further stabilizing the portal-capsid protein complex. The complex then recruits additional gp23 molecules through portal morphing, as well as gp23-gp23 interactions, assembling five gp23 capsomers encircling the portal hub. With the capsomers precisely oriented, this nucleating complex would then drive rapid assembly by channeling the abundantly-expressing gp23 molecules into the growing capsid shell. Previous genetic studies suggest that the major core protein gp22 is also a part of the nucleation complex^9,31^. Although it is unknown which region of the portal interacts with gp22, the crown domain, portal’s most capsid-internal part, is a strong candidate. The shell and the core then co-assemble to generate a prohead of precise dimensions. A shortened whisker would make the initial gp23 capture inefficient, as was observed in our genetic experiments where the 4-del mutant produced fewer proheads and was lethal. Further, the fidelity of portal-capsid interactions was compromised, resulting in the assembly of many round particles and loss of size control over the triangulation number.

It is reasonable to suggest, and there are indications from our biochemical data, that portal morphing might also be important for capsid expansion and DNA packaging. Docking of a pentameric packaging motor at the clip domain of the portal^32,40,41^, which creates another symmetry-mismatch, might induce conformational changes in the portal, causing portal morphing again by remodeling portal’s interactions with the neighboring gp23* subunits in the symmetry-mismatched vertex. This in turn might induce conformational changes in the gp23 subunits and propagating these changes throughout the capsid shell causing capsid expansion. There is evidence that capsid expansion begins at the portal and there are also mutants in the portal protein that are defective in expansion^42^. During DNA translocation, the motor subunits fire one at a time to translocate ~2 bp of DNA^4–6^, which might also involve portal morphing to make the firing subunit a “special” subunit through its unique interaction with a portal subunit(s)^4,43^.

Analysis of the recently published, in situ 12-fold-averaged cryo-EM reconstructions of the portals of phages phi29^16^, P23-45^17^, P68^44^, and herpes simplex virus 1^15^, suggests that the portal morphing likely occurs in many other viruses. Because of the 12-fold averaging, these reconstructions can resolve only those parts of the portal structure, which obey the 12-fold symmetry, whereas the symmetry-mismatched capsid-portal interface is not resolved. We found that the N-terminal portal regions are not resolved in all these reconstructions. Also, in the P68 portal^44^, there is the long unresolved loop formed by residues 84–103, located in the periphery of the wing domain. Since these unresolved portal regions do not obey the 12-fold symmetry, they probably interact with the capsid and morph to compensate for the symmetry mismatch. Notably, in these structures the morphing might occur not only in the unresolved portal regions, but also in some other regions with interpretable 12-fold-averaged density. This might happen if only a few out of the 12 portal subunits morph, deviating from a predominant major conformation. In such case, the averaged cryo-EM density would be dominated by the major conformation and, therefore, would be interpretable.

Structural morphing of portal, thus, represents an extraordinary viral adaptation, evolved to create a dynamic symmetry-mismatched vertex that is fundamentally essential to direct various key assembly processes involved in building an infectious virus particle. It allows the interacting components to maintain distinct structures and symmetries while enabling them to conduct inter-structural transactions that are essential for function. This might be a general phenomenon in viruses and numerous structurally plastic macromolecular assemblies in biological systems.

## Methods

### Phage T4 capsids

Phage T4 capsids were prepared from *E. coli* P301 (suppressor-minus) cells infected with the neck-less, tail-less *10am.13am* mutant phage, as described previously^34,45^. These are mature emptied capsids because the encapsidated DNA is released in the absence of the gp13 neck protein and then digested using Benzonase (DNAse I). For preparation of empty proheads using the 4-del portal protein mutant, the gp20 portal protein in which the amino acids 2–5 were deleted was expressed in *E. coli* BL21 (DE3) (suppressor-minus) and infected with *10am.13am.17am.20am*E481 phage. Because this mutant also lacks the packaging motor protein gp17, DNA packaging is not initiated, resulting in the accumulation of empty proheads. Control wild-type (WT) empty proheads containing the WT portal protein were prepared in the same way by using the BL21 (DE3) cells expressing the full-length gp20.

The empty capsids were purified from the lysates of infected cells by differential centrifugation, CsCl density gradient centrifugation, and ion-exchange chromatography, according to the procedures described previously^34,45^. Briefly, after low speed (4300 × *g* for 10 min) and high speed (34,500 × *g* for 45 min) centrifugations, the samples were layered on a 5 ml cesium chloride gradient and centrifuged at 34,000 × *g* for 1 h in a SWTi55 rotor. The heads form a band near about half the length of the tube. The band was extracted and dialyzed overnight against 10 mM Tris-HCl pH 7.5, 50 mM NaCl, and 5 mM MgCl_2_. The capsids were then purified by ion-exchange chromatography on a DEAE-Sephacel column using a linear gradient of 50–300 mM NaCl. Various peak fractions containing the head particles were concentrated by Amicon membrane filter (30 K cut-off) to about 10^10^ particles/µl, flash-frozen with liquid nitrogen, and stored at −80 °C.

### Cryo-EM data collection

About 3 μl aliquots of the emptied mature T4 capsids prepared as above were frozen onto Lacey carbon EM grids using a Gatan CP3 freezer with a blotting time of 6 s. The EM grids were then loaded on an FEI Titan Krios EM operated at 300 kV and equipped with a Gatan K2 Summit detector. The data collection was performed using the Leginon program^46^ with a magnification of 14,000  (physical pixel size: 2.08 Å) in the “super-resolution” mode, which resulted in a pixel size of 1.04 Å per pixel. The dose rate was ~10 e^−^/(pixel·s). A total of 4377 movies, each composed of 40 frames, were collected. Each frame has an exposure time of 250 ms.

### Cryo-EM data processing

The movies were subjected to motion correction and dose weighting using the MotionCorr2 program^47^. Micrographs were produced by summing up the aligned and dose-weighted frames (excluding the first frame) of each movie. To accelerate image processing, the pixel size of the micrographs was rescaled to 1.44 Å per pixel using the relion_preprocess function^48^. The contrast transfer function (CTF) parameters estimation, particle picking and 3D classification were performed using the cisTEM program^49^.

A total of 53,608 particles were picked and selected for further processing using automatic picking followed by manual adjustment. The previously reported 10-Å resolution, 5-fold averaged cryo-EM reconstruction of the emptied mature prolate T4 capsid^7^, low-pass filtered to 40 Å, was used as an initial model for determining the initial alignment parameters of each particle using the jspr program^50^. The cryo-EM reconstruction was initially refined assuming D5 symmetry. The symmetry was then relaxed to C5, resulting in a 4.3 Å-resolution reconstruction.

To resolve the symmetry mismatch between the portal (C12) and the capsid (C5), the previously described sub-particle reconstruction and symmetry relaxation procedures^15,51,52^ were employed as follows. The alignment and CTF parameters of each sub-particle around the portal vertex were recalculated based on the corresponding values of their original particles (or “parental particles”), previously determined when calculating the 5-fold-symmetric reconstruction of the capsid. The alignment parameters and defocus values of the sub-particles were further refined by assuming the C5 symmetry, resulting in a 3.4 Å-resolution, 5-fold-averaged reconstruction of the gp23* capsomers surrounding the portal. The previously imposed 5-fold symmetry were then expanded by using the symmetry-expansion procedure in Relion^53^. Sub-particles were extracted from the parental particles using the Relion program^48^. A round of 3D classification was performed by fixing the alignment parameters of each sub-particle, applying a soft mask around the portal assembly and assuming C12 symmetry. This resulted in five nearly identical reconstructions with approximately 72° rotational differences. The particle occupancies of each class were ~20%. One out of the five classes was chosen for further refinement. Final particle assignment for this class was decided by comparing the occupancy values among each set of five sub-particles that originate from the same parental particle. Further refinement using the cisTEM program^49^ by assuming C12 and C1 symmetry gave us two reconstructions at resolutions of 3.8 Å and 4.5 Å, respectively. Soft masks around the portal protein and the portal vertex were applied when refining the C12 and C1 reconstructions, respectively. The resolutions of all these cryo-EM reconstructions were determined by the gold-standard FSC cutoff of 0.143^33^.

### Model building and refinement

The 3.4 Å-resolution, 5-fold-averaged reconstruction of the gp23* capsomers around the portal was interpreted by fitting individual capsid subunits derived from the previously reported cryo-EM structure of the empty mature isometric T4 capsid^34^ into the density map. The fitted model was then rebuilt using the Coot program^54^ and refined using Rosetta^55^. The 3.8 Å-resolution in situ 12-fold-averaged reconstruction of the portal protein, gp20, was initially interpreted by fitting the previously reported structure of the recombinantly expressed T4 portal lacking its 73 N-terminal residues^7^ into the density map. The fitted structure was then rebuilt using the Coot program^54^ and refined using the Rosetta program^55^. The in situ asymmetric structure of the portal vertex was initially modeled by fitting the in situ 12-fold-averaged structure of gp20 and the 5-fold-averaged structure of the surrounding gp23* capsomers into the 4.5 Å-resolution asymmetric reconstruction. The asymmetric structure was then rebuilt in Coot and refined using Rosetta.

### Mutagenesis: CRISPR editing and recombinational rescue

Mutations were introduced into the portal protein gene 20 (*g20*) using our recently described CRISPR genome editing strategy^56^. Recombinant *E. coli* were constructed by transforming *E. coli* BL21 with two plasmids, a CRISPR-Cas plasmid containing a spacer sequence corresponding to the site to be mutated, and a donor plasmid containing the desired mutation flanked by ~500-bp homologous sequences on either side of the mutant sequence. The mutations include single or multiple amino acid substitutions, one to four amino acid deletions, double amber mutations, all in the N-whisker sequence. Upon infection of these *E. coli* with WT T4 phage (37 °C), the injected phage genome is cleaved at the proto-spacer sequence, the site where mutation is to be introduced, by the CRISPR-Cas9 genome editing complex. Recombination of the cleaved ends with the homologous flanking sequences in the donor plasmid transfers the mutation into T4 genome resulting in the rescue of the otherwise Cas9-inactivated genome. If the mutation retains function, plaques will appear from the rescued genome. Individual plaques were then purified and sequenced to confirm that the genome contained the desired mutation. If the mutation results in functional defects, plaques will not appear even though the genome would be rescued. *E. coli* containing only the respective CRISPR-Cas9 plasmid but lacking the donor plasmid were used as a control in each experiment. Various spacer-containing CRISPR-Cas plasmids and donor plasmids containing deletions in the N-terminal whisker of *g20* were constructed to determine the functional significance of the deleted whiskers. For construction of single or triple amino acid combinatorial libraries, the nucleotide sequence corresponding to the amino acid(s) was randomized in the primers used to construct the donor plasmid and the library of plasmids was transformed into *E. coli* BL21. Consequently, each transformant carries one of the mutations at the respective amino acid(s). Testing a large number of colonies by the above genetic rescue strategy resulted in an unbiased screening of the library to determine the functional importance of the mutated amino acid(s). A number of mutants were sequenced to connect the change in the nucleotide (amino acid) sequence to the phenotype.

### Mutagenesis: rescue of amber phage mutants in N-whisker

The codons for amino acids Lys2 and Asn4 of N-whisker were replaced by amber termination codons using the CRISPER mutagenesis strategy described above. Among the recovered progeny phage, the mutant phages that formed plaques on the amber suppressor *E. col*i B40 (*sup*^1^) but not on suppressor-minus *E. coli* P301 (*sup*^*-*^) were selected and sequenced to confirm the presence of amber mutations. Several independent mutant phage isolates containing the Lys2*am*-Asn4*am* mutations were tested for rescue by plating on *E. coli* BL21 (*sup*^*-*^) carrying *g20* donor plasmid DNAs containing one, two, three, or four amino acid deletions. *E. coli* BL21 containing WT *g20* or the empty vector lacking *g20* insert were used as positive and negative controls, respectively. Plaques were produced only in one or two amino acid deletions, whereas no phages were recovered from three or four amino acid deletions. Phage were purified from individual plaques by serial dilution and were re-tested to confirm the phenotype, as well as sequenced to confirm the transfer of the respective deletion into phage genome.

### Reporting summary

Further information on research design is available in the Nature Research Reporting Summary linked to this article.

## Supplementary information

Supplementary Information

Reporting Summary

Description of Additional Supplementary Files

Supplementary Movie 1

Supplementary Movie 2

Supplementary Movie 3

## Data Availability

The cryo-EM reconstructions calculated using C1, C12, and C5 symmetry have been deposited in the Electron Microscopy Data Bank under the accession codes EMD-20956, EMD-20960, and EMD-20961, respectively. The coordinates of the portal protein and five surrounding capsomers have been deposited to the Protein Data Bank under the accession code PDB 6UZC [10.2210/pdb6UZC/pdb]. Other data that support the findings of this study are available from the authors upon request.
