# Supplementary material for: Structural morphing in a symmetry-mismatched viral vertex
Source: Nat Commun. 2020 Apr 6;11:1713. doi: 10.1038/s41467-020-15575-4 (PMC7136217; doi:10.1038/s41467-020-15575-4)
Supplement: Supplementary file 1 — Supplementary Information [file 41467_2020_15575_MOESM1_ESM.pdf]

Structural morphing in a symmetry-mismatched viral vertex

Supplementary Information

Qianglin Fang et al.

Supplementary Figures and Figure Legends

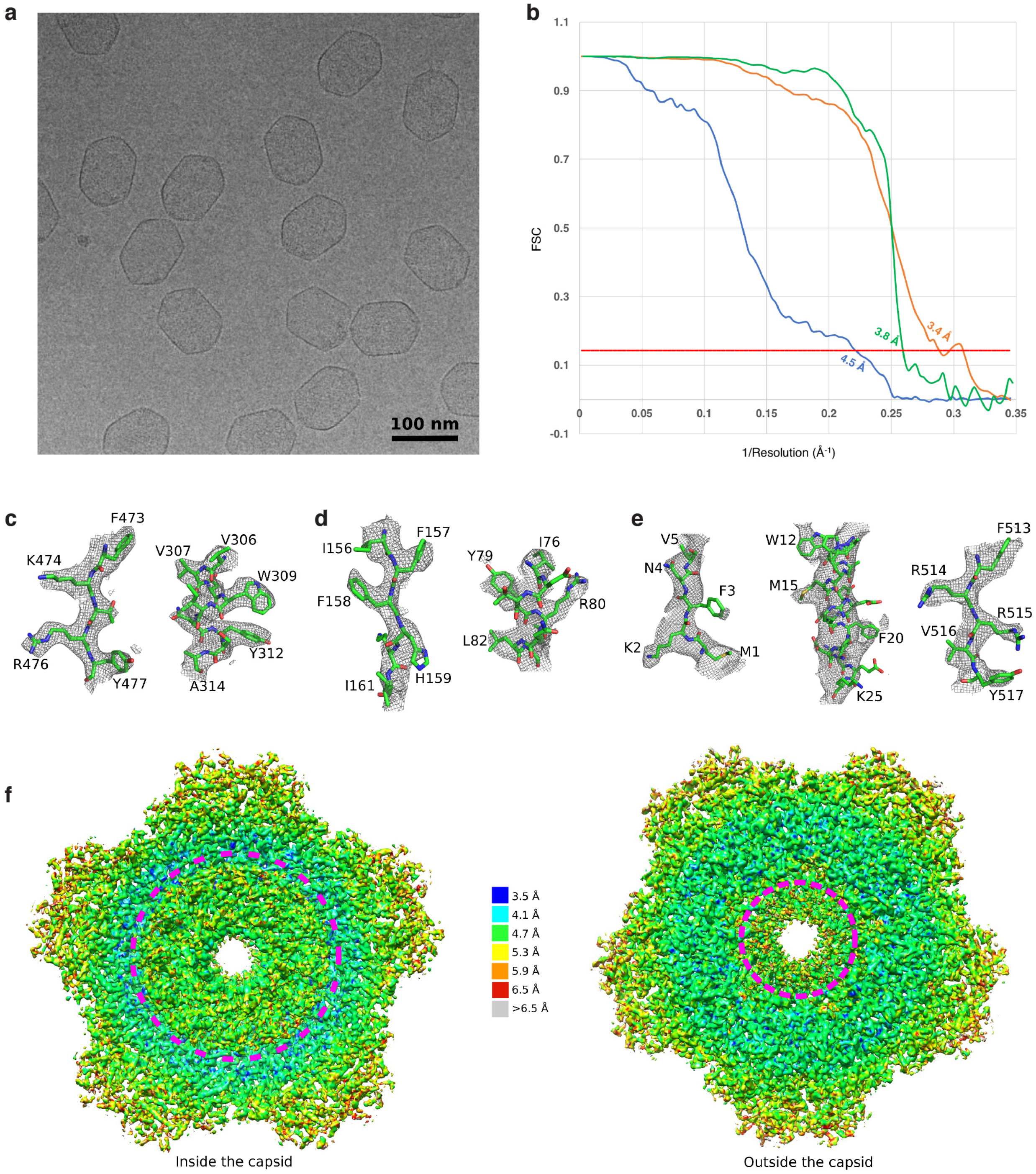

Supplementary Figure 1. Cryo-EM reconstructions. a, EM image of empty T4 capsids. **b,** FSC curves. The curves of the 12-fold-averaged reconstruction of the portal assembly, the 5-fold-

averaged reconstruction of the gp23* capsomers surrounding the portal assembly, and the asymmetric reconstruction of the portal and surrounding capsomers are colored in green, orange and blue, respectively. **c,** Cryo-EM density of selected regions in the 5-fold-averaged reconstruction of the gp23* capsomers surrounding the portal assembly. **d,** Cryo-EM density of selected regions in the 12-fold-averaged reconstruction of the portal protein. **e,** Cryo-EM density of selected regions in the asymmetric reconstruction of the portal and surrounding capsomers. **f,** Local resolution of the asymmetric reconstruction of the portal and surrounding capsomers estimated using the ResMap program[1]. The portal region is outlined by magenta dashed circles.

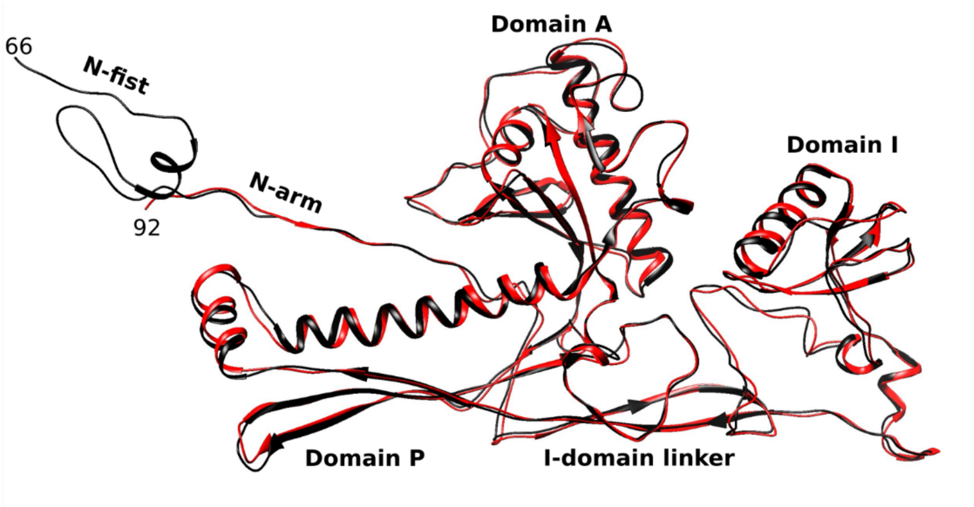

Supplementary Figure 2. Atomic model of gp23 D1 subunit from the *in situ* structure (red) superimposed with a similarly located "distal" gp23 subunit surrounding the gp24 capsomer in the icosahedrally-averaged head structure (black, PDB: 5VF3). Individual domains of gp23 are labeled as in Chen *et al.*[2].

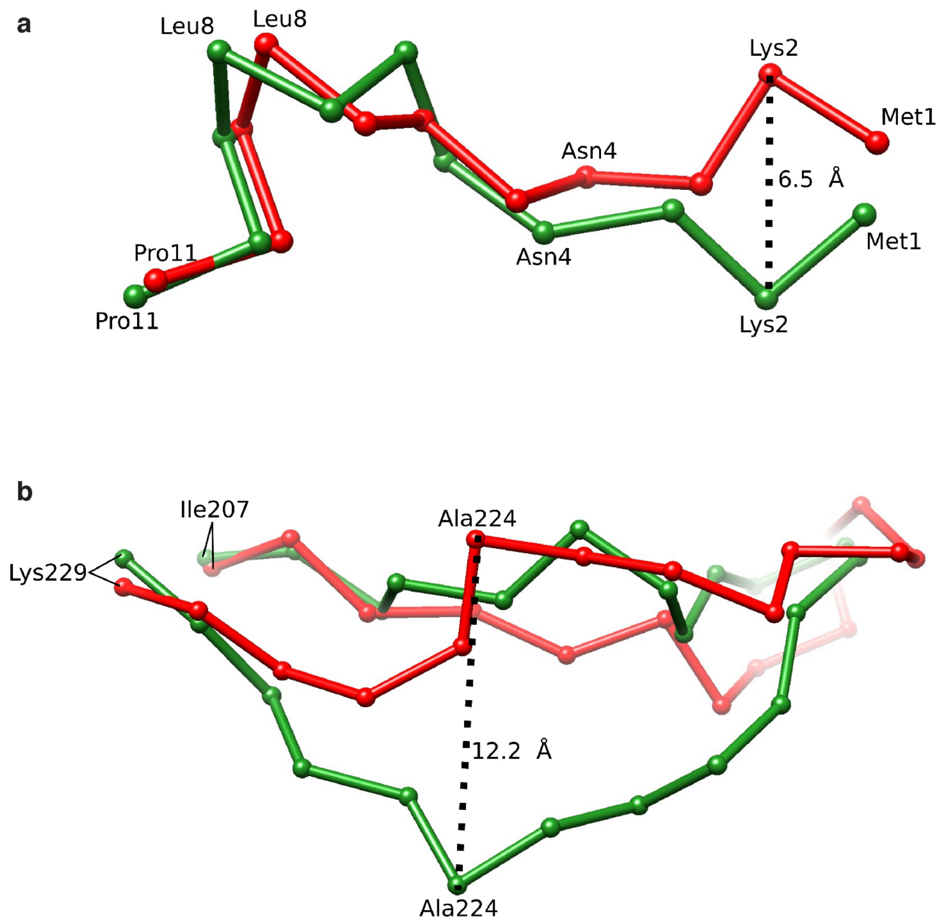

Supplementary Figure 3. Large conformational differences in some regions of the portal subunits that are involved in forming the portal-capsid interface. a, The N-whisker regions. The portal subunits 1 (green) and 6 (red) are superposed. The Cα traces are shown. **b,** The loop regions Asp207-Lys229. The portal subunits 3 (red) and 7 (green) are superposed.

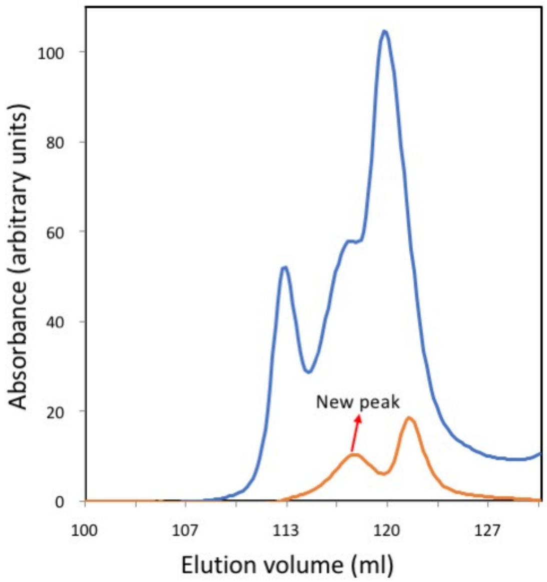

Supplementary Figure 4. Elution profiles of WT (blue) and 4-del mutant (orange) proheads. The proheads were prepared as described in Methods. The first peak in the elution profile of WT proheads corresponds to expanded proheads that are missing in the 4-del mutant and the second peak (with its shoulder) corresponds to unexpanded proheads. A new peak corresponding to partially expanded proheads and round heads of various sizes appears in the 4-del mutant.

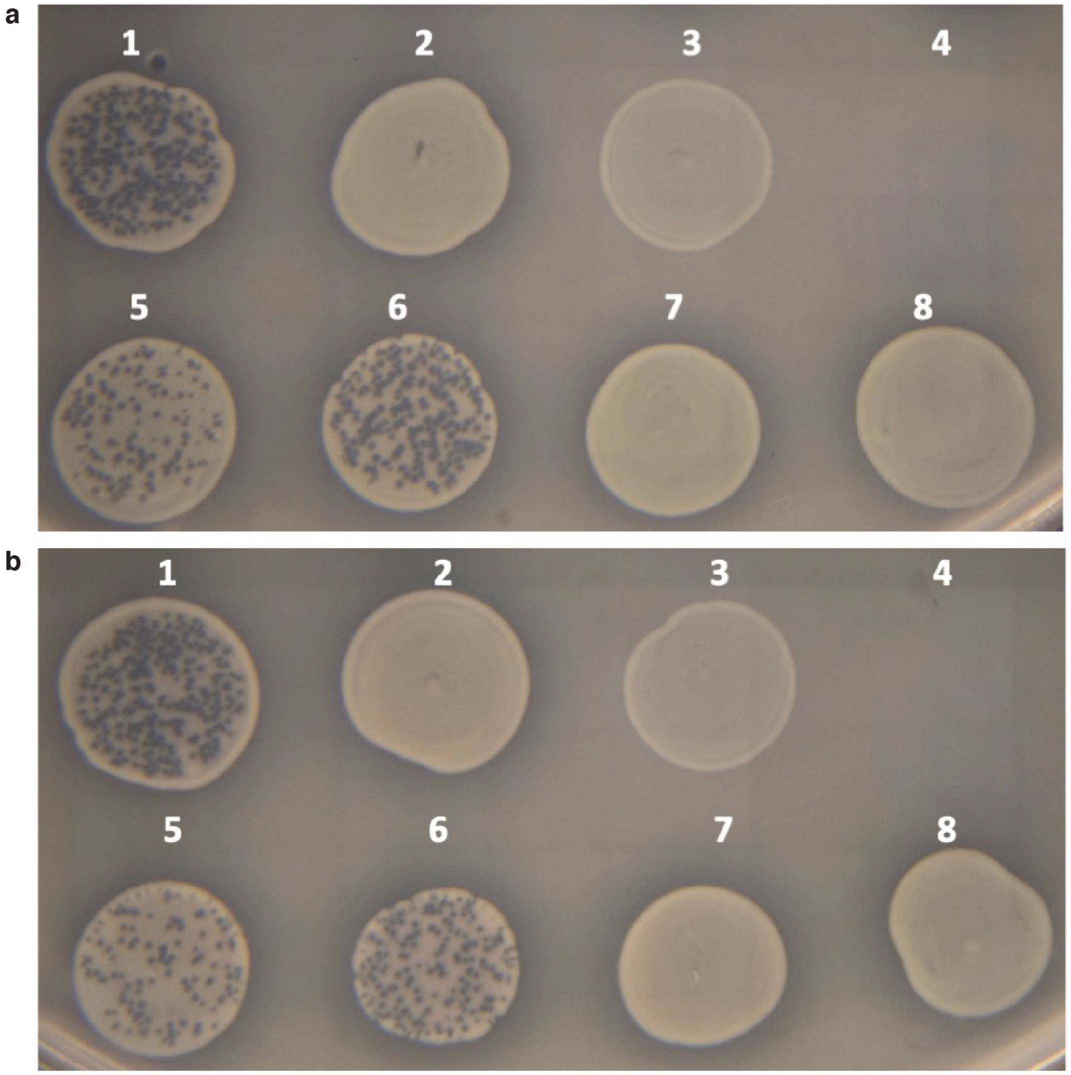

Supplementary Figure 5. Recombinational rescue of amber mutations in N-whisker of gene 20 by various deletion mutants in N-whisker. *E. coli* BL21 (*sup*$^-$) bacteria containing *g20* donor plasmids with WT (1), one (5), two (6), three (7), or four (8) amino acid deletions in N-whisker were spotted on a lawn of *E. coli* P301 (*sup*$^-$). In addition, *E. coli* BL21 bacteria with (2) or without (3) empty plasmid vector (2), or the Lys2-Asn4 double amber T4 mutant phage (4) were also spotted in the same plate to verify that there was no background phage in the negative controls. About 5×10^4 amber mutant phages were spotted on top of the *E. coli* BL21 spots (1-3 and 5-8) and the plates were incubated at 37 °C overnight. Plaques would appear only if the

deletion mutant rescued the amber mutations by recombinational exchange. The *E. coli* BL21 containing WT *g20* sequence or empty plasmid served as positive and negative controls, respectively. **a** and **b** correspond to two independent isolates of Lys2-Asn4 double amber mutants.

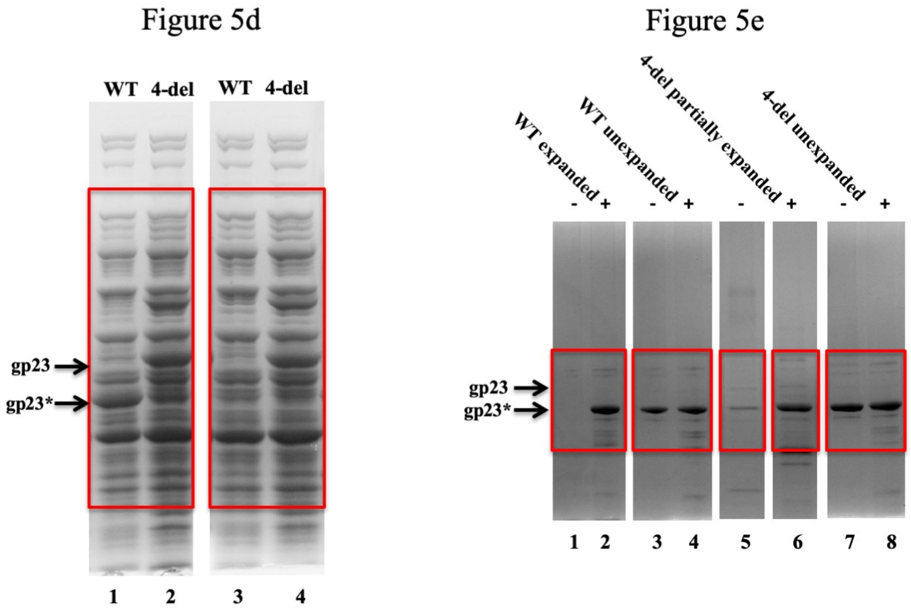

Supplementary Figure 6. Uncropped protein gels of panels d and e shown in Fig. 5. Red boxes correspond to the areas shown in the respective panels in Fig. 5 of the main paper.

Supplementary Table 1

Cryo-EM data collection, refinement and validation statistics

**Map**	C1 map	C5 map	C12 map
**Data collection and processing**			
Microscope	FEI Titan Krios	FEI Titan Krios	FEI Titan Krios
Magnification	14,000	14,000	14,000
Voltage (kV)	300	300	300
Detector	Gatan K2 Summit	Gatan K2 Summit	Gatan K2 Summit
Recording mode	Super-resolution	Super-resolution	Super-resolution
Dose rate (e^-/(pixel·s))	10	10	10
Frame exposure time (ms)	250	250	250
Movie micrograph exposure time (s)	10	10	10
Total dose (e^-/Å^2)	23	23	23
Defocus range (μm)	1.0-4.0	1.0-4.0	1.0-4.0
Pixel size (Å)	1.04	1.04	1.04
Symmetry imposed	C1	C5	C12
Map resolution (FSC 0.143; Å)	4.5	3.4	3.8
Map sharpening B-factor (Å^2)	-180	-180	-176
**Structure building and Validation**			
R.m.s. deviations			
Bond lengths (Å)	0.018		
Bond angles (°)	1.776		
MolProbity score	1.74		
Clashscore	6.47		
Poor rotamers (%)	0.26		
Ramachandran plot			
Favored (%)	94.44		
Allowed (%)	4.96		
Disallowed (%)	0.60		

Supplementary References

1. Kucukelbir, A., Sigworth, F.J. & Tagare, H.D. Quantifying the local resolution of cryo-EM density maps. *Nat. Methods* **11**, 63-65 (2014).
2. Chen, Z. et al. Cryo-EM structure of the bacteriophage T4 isometric head at 3.3-Å resolution and its relevance to the assembly of icosahedral viruses. *Proc. Natl. Acad. Sci. U.S.A.* **114**, E8184-E8193 (2017).
