# Supplementary material for: Structural morphing in a symmetry-mismatched viral vertex
Source: Nat Commun. 2020 Apr 6;11:1713. doi: 10.1038/s41467-020-15575-4 (PMC7136217; doi:10.1038/s41467-020-15575-4)
Supplement: Supplementary file 3 — Description of Additional Supplementary Files [file 41467_2020_15575_MOESM3_ESM.docx]

Description of Additional Supplementary Files

File Name: Supplementary Movie 1

Description: The overall structure of the symmetry-mismatched viral portal vertex. The dodecameric portal assembly surrounded by five capsomers is shown.

File Name: Supplementary Movie 2

Description: Morphing of portal protein subunits for forming the same charge interactions with the capsid protein in different structural environments. The morphing of Glu66 of portal protein subunit 1 to that of portal protein subunit 6 is shown.

File Name: Supplementary Movie 3

Description: Morphing of N-whisker to form methionine-metal clusters with the same amino acid residues of the capsid protein. The morphing of the N-whisker of portal protein subunit 1 to that of portal protein subunit 6 is shown.
